# CT-guided fine needle aspiration cytology diagnosis of extra-adrenal pheochromocytoma

**DOI:** 10.4103/0970-9371.66689

**Published:** 2010-01

**Authors:** M Rangaswamy, Sandeep P Kumar, M Asha, GV Manjunath

**Affiliations:** Department of Pathology, JSS Medical College, JSS University, Mysore, India

**Keywords:** CT-guided FNAC, retroperitoneal, pheochromocytoma, paraganglia

## Abstract

Pheochromocytoma is a rare tumor, accounting for <0.1% of the hypertensive population. Extra-adrenal pheochromocytomas (EAPs) are rarer still, accounting for 10% of all pheochromocytomas. Pheochromocytomas are functional catecholamine-secreting tumors of the paraganglionic chromaffin cells found in the adrenal medulla and the extra-adrenal paraganglia cells. EAPs are readily detected by computed tomography (CT) as soft tissue masses closely associated with the entire length of the abdominal aorta. Here, we present a rare case of EAP in a 45-year-old male hypertensive patient diagnosed by CT-guided fine needle aspiration cytology. The smears showed loosely cohesive tumor cells with prominent anisokaryosis and abundant eosinophilic, granular cytoplasm. The diagnosis was later confirmed by histopathology. The present case also highlights the fact that fine needle aspiration of pheochromocytoma is not necessarily contraindicated.

## Introduction

Extra-adrenal pheochromocytomas (EAPs) are uncommon neuroendocrine cell neoplasms arising from the paraganglia, which are widely dispersed throughout the body in small nests, from the upper cervical region to the floor of the pelvis, adjacent to the autonomic nervous system and their ganglia.

Fine needle aspiration cytology (FNAC) of EAPs has been reported to have a variety of morphologic patterns that can result in a misdiagnosis because these lesions can mimic a wide spectrum of epithelial and mesenchymal tumors.[1]

Fine needle aspiration of clinically suspected pheochromocytoma is generally regarded as a contraindication in view of the risk of precipitating a hypertensive crisis. However, this complication occurs only rarely as per the literature.[2]

## Case Report

**Figure 1:**
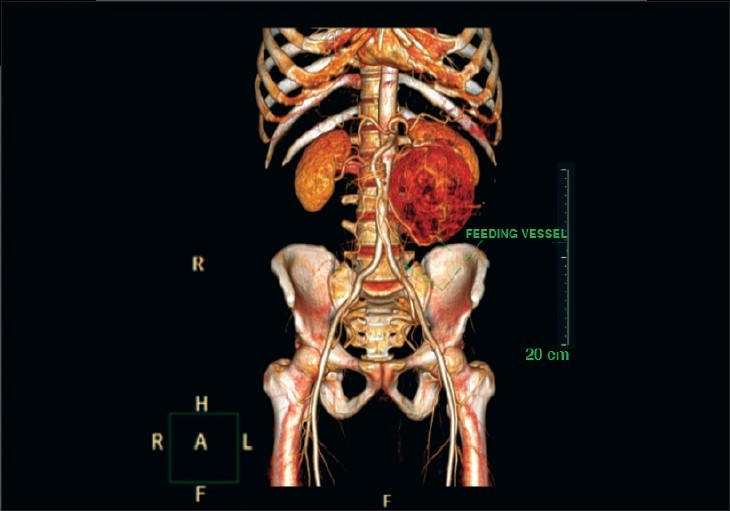
Computed tomography angiogram showing a tumor situated in front of the left kidney. The left kidney and adrenals are normal

A 45-year-old male presented with an asymptomatic abdominal mass since one year. The patient was a known hypertensive since six months. Both ultrasonography and computed tomography (CT) revealed a 12 cm × 10 cm retroperitoneal soft-tissue tumor situated anteromedial to the lower part of the left kidney [Figure 1].

FNA was performed under CT guidance using a 22-gauge needle. The aspirated material was spread on glass slides. Air-dried and 95% ethyl alcohol-fixed slides were stained by may-grünwald giemsa, hematoxylin and eosin and Papanicolaou stains. The remaining material from the needle hub was fixed in 65% alcohol and sent for cell block.

**Figure 2:**
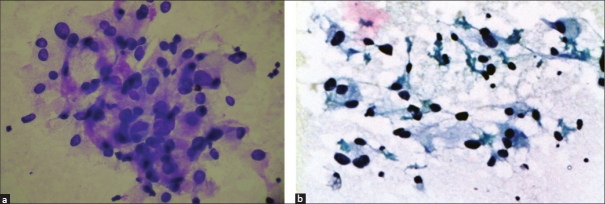
(a) A cluster of tumor cells showing indistinct cell borders, abundant granular eosinophilic cytoplasm and anisokaryosis (H and E, ×400). (b) Occasional binucleate and spindle cells are seen (PAP, ×400)

**Figure 3:**
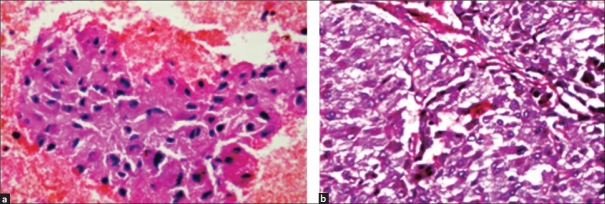
(a) Cell block showing classical features of pheochromocytoma (H and E, ×400). (b) Tissue section shows tumor cells arranged in the characteristic Zellballen pattern (H and E, ×400)

FNA smears showed loosely cohesive clusters and scattered tumor cells with prominent anisokaryosis, abundant eosinophilic granular cytoplasm and indistinct cell borders. Occasional binucleate cells were seen [Figure 2a and 2b]. On the basis of clinical presentation, CT findings and FNA features, a differential diagnosis of EAP was strongly thought of. Cell block showed features consistent with pheochromocytoma [Figure 3a]. Urinary vanillyl mandelic acid (VMA) levels were elevated.

After adequate pre-operative precautions, the patient was taken for surgery. At surgery, a retroperitoneal, solitary, well-encapsulated tumor of size 15 cm × 12 cm was seen in front of the left kidney and was encasing the left renal vessels. The left kidney and adrenals were normal. The tumor was excised completely.

Histologically, it was a well-encapsulated tumor with cells arranged in a Zellballen pattern. Individual cells were large, polygonal to spindle-shaped, having abundant granular, eosinophilic cytoplasm and vesicular nuclei with prominent nucleoli. Areas of hemorrhage were noted. The histology was compatible with pheochromocytoma [Figure 3b]. Postoperatively, the urinary VMA levels returned to normal.

## Discussion

Pheochromocytomas are uncommon neoplasms composed of chromaffin cells that synthesize and release catecholamines and, in some instances, peptide hormones. These tumors are important because they give rise to surgically correctable forms of hypertension.[3]

EAPs have been reported to occur in a wide variety of sites and generally parallel the course of the sympathetic nervous system.[1] They occur at the para-adrenal area in over 37.5% of the patients and in the organ of Zuckerkandl at the aortic bifurcation in 12.5% of the cases.[4] In the present case, the tumor was located in the region of the organ of Zuckerkandl.

Approximately 10% of EAPs, especially retroperitoneal ones, can behave in a malignant fashion, as proven by the presence of metastatic lesions. Accurate localization in a metastatic lesion may not be as helpful diagnostically as that for a primary EAP. The common locations reported for metastases are liver, bone, lymph node and lung.[1]

FNA has proven to be a valuable tool in the diagnosis of mass lesions. However, its use in the diagnosis of EAPs is controversial due primarily to the potential risk of hemorrhage within the lesion, which can be life-threatening. Consequently, there have been relatively few reports discussing the cytologic features of EAP. Nevertheless, when EAP is clinically unsuspected due to the lack of a typical presentation, FNA is often the modality used for the initial work-up.[1]

Although FNA cytology is helpful in the diagnosis of these tumors, there is a significant cytomorphologic overlap with other tumors. Unusual cytologic features of EAP should be kept in mind. Cells with prominent nucleoli may simulate malignant glandular cells and suggest adenocarcinoma. Dense, often eosinophilic cytoplasm may be observed in EAP, suggesting squamous differentiation. Neuroendocrine tumors tend to display prominent nuclear atypia and pleomorphism. In fact, this feature has been used as a soft criterion to distinguish paraganglioma from adenocarcinoma since adenocarcinoma rarely shows such tremendous cellular pleomorphism within the same tumor. The exact anatomic location of the tumor is crucial to an accurate cytologic diagnosis on a FNA specimen.[1]

The presence of hypertension with symptoms of adrenergic excess should alert the clinician to the tumors of adrenal medulla as well as the extra-adrenal paraganglion system.[4] Urinary estimation of catecholamine and their metabolites is a better biochemical investigation than plasma estimation for the diagnosis of these lesions. CT scan is the imaging modality of choice for localization. I^131^ - or I^123^ - labeled meta-iodobenzylguanidine scan is indicated in extra-adrenal tumors to rule out multicentricity and metastases.[5]

Surgery after adequate pre-operative preparation remains the treatment of choice. A life-long follow-up is indicated as the EAPs are more likely to recur and to metastasize. Annual determination of urinary catecholamines and their metabolites is recommended. Persistent hypertension after successful removal of pheochromocytoma occurs in approximately 25% of the cases.[5]

In conclusion, the cytologic features of EAP, although suggestive, are not specific. A high index of suspicion and knowledge of clinical information, exact anatomic location and cytologic morphology combined with appropriate ancillary studies are the key to an accurate diagnosis.[1] FNA of pheochromocytoma is not necessarily contraindicated but aspiration must be performed in an area equipped with the therapeutic tools necessary to control a pheochromocytoma crisis.[6]
